# Algorithms to Improve Fairness in Medicare Risk Adjustment

**DOI:** 10.1001/jamahealthforum.2025.2640

**Published:** 2025-08-29

**Authors:** Marissa B. Reitsma, Thomas G. McGuire, Sherri Rose

**Affiliations:** 1 Department of Health Policy, School of Medicine, Stanford University, Stanford, California; 2 Department of Health Care Policy, Harvard Medical School, Boston, Massachusetts

## Abstract

**Importance:**

Payment system design creates incentives that affect health care spending, access, and outcomes. With Medicare Advantage accounting for more than half of Medicare spending, changes to its risk adjustment algorithm have the potential for broad consequences.

**Objective:**

To assess the potential for algorithmic tools to achieve more equitable plan payment for Medicare risk adjustment while maintaining current levels of performance, flexibility, feasibility, transparency, and interpretability.

**Design, Setting, and Participants:**

This diagnostic study included a retrospective analysis of traditional Medicare enrollment and claims data generated between January 1, 2017, and December 31, 2020, from a random 20% sample of non–dual-eligible Medicare beneficiaries with documented residence in the US or Puerto Rico. Race and ethnicity were designated using the Research Triangle Institute enhanced indicator. Diagnoses in claims were mapped to hierarchical condition categories. Algorithms used demographic indicators and hierarchical condition categories from 1 calendar year to predict Medicare spending in the subsequent year. Data analysis was conducted between August 16, 2023, and January 27, 2025.

**Main Outcomes and Measures:**

The main outcome was prospective health care spending by Medicare. Overall performance was measured by payment system fit and mean absolute error. Net compensation was used to assess group-level fairness.

**Results:**

The main analysis of Medicare risk adjustment algorithms included 4 398 035 Medicare beneficiaries with a mean (SD) age of 75.2 (7.4) years and mean (SD) annual Medicare spending of $8345 ($18 581); 44% were men; fewer than 1% were American Indian or Alaska Native, 2% were Asian or Other Pacific Islander, 6% were Black, 3% were Hispanic, 86% were non-Hispanic White, and 1% were part of an additional group (termed as other in the Centers for Medicare & Medicaid Services data). Out-of-sample payment system fit for the baseline regression was 12.7%. Constrained regression and postprocessing both achieved fair spending targets while maintaining payment system fit (constrained regression, 12.6%; postprocessing, 12.7%). Whereas postprocessing increased mean payments for beneficiaries in minoritized racial and ethnic groups (American Indian or Alaska Native, Asian or Other Pacific Islander, Black, and Hispanic individuals) only, constrained regression increased mean payments for beneficiaries in minoritized racial and ethnic groups and beneficiaries in other groups residing in counties with greater exposure to socioeconomic factors that can adversely affect health outcomes.

**Conclusions and Relevance:**

Results of this study suggest that constrained regression and postprocessing can incorporate fairness objectives into the Medicare risk adjustment algorithm with minimal reduction in overall fit. These feasible changes to the Medicare risk adjustment algorithm could be considered by policymakers aiming to address health care disparities through payment system reform.

## Key Points

**Question:**

Is use of modern algorithms associated with improved group-level fairness while maintaining overall performance in Medicare risk adjustment?

**Findings:**

In this diagnostic study including 4.4 million Medicare beneficiaries of Medicare risk adjustment algorithms, constrained regression and postprocessing both achieved fair spending targets for minoritized racial and ethnic groups without compromising overall payment system fit.

**Meaning:**

These findings suggest that feasible extensions of the current risk adjustment algorithm can yield additional payments for minoritized racial and ethnic groups in Medicare.

## Teaser

This diagnostic study evaluates 2 algorithmic approaches to risk adjustment to achieve Medicare payments more equitable to minoritized racial and ethnic groups.

## Introduction

Risk adjustment is a core component of capitated payment systems aiming to mitigate selection incentives that encourage plans to attract profitable enrollees and avoid unprofitable enrollees.^1,2,3,4,5,6,7^ The algorithms used to risk adjust payments to Medicare Advantage plans predict health care spending as a function of beneficiary demographic characteristics and a set of diagnosed health conditions.^1,5,8^ With Medicare Advantage accounting for more than half of Medicare spending, improvements to Medicare’s risk adjustment system have the potential to lead to substantial changes in health care spending, access, and outcomes.

Currently, Medicare Advantage plan payment risk adjustment is based on least squares regression. Prior studies have proposed approaches to improve risk adjustment by increasing predictive power, mitigating incentives for upcoding, and reducing opportunities for favorable selection.^9,10,11,12,13,14,15,16,17,18,19,20^ These studies use a range of methods, including constrained regressions, penalized regressions, data transformations, random forests, and other machine learning algorithms. Only a few consider fair regression methods, which optimize for both overall and group-level fit metrics for marginalized groups. These methods do not add marginalized groups as predictors in the regression, which can reinforce inequities from the data.^14,16^ Notably, algorithms to achieve fairness goals across multiple minoritized racial and ethnic groups (American Indian or Alaska Native, Asian or Other Pacific Islander, Black, and Hispanic individuals) in Medicare have not been examined previously. This gap in the literature is important because a greater percentage of Asian or Other Pacific Islander, Black, and Hispanic beneficiaries are enrolled in Medicare Advantage plans compared with White beneficiaries.^21,22^ Additionally, the population aged 65 years and older is projected to become more racially and ethnically diverse over the coming decades.^23^

Although Medicare eligibility reduces racial and ethnic disparities in insurance coverage, disparities in health care access, use, spending, and outcomes persist.^24,25,26,27,28,29^ Historical fee-for-service spending data, which are used by the Centers for Medicare & Medicaid Services (CMS) to estimate risk scores and determine payments, embed these long-standing disparities. In a 2023 study, risk-adjusted payments were documented to exceed observed spending for Black and Hispanic beneficiaries. This suggests that population-based payment systems could promote health equity through resource reallocation toward historically marginalized groups.^28^ Access and use disparities for Medicare Advantage beneficiaries remain in the existing payment system,^22,30^ which can lead to risk-adjusted payments exceeding the observed (suboptimal) spending.

This study assesses the potential for algorithmic tools to achieve more equitable plan payment for Medicare. First, we described existing levels of net compensation by racial and ethnic groups and propose a basic measure of health care spending disparity that can inform fair spending targets. We then evaluated 2 approaches to achieve fair spending targets: constrained regression and postprocessing. Our analysis presents algorithms for Medicare risk adjustment that promote more equitable payments, maintain current levels of performance, and remain flexible, feasible, transparent, and interpretable.

## Methods

### Study Design and Data

In this diagnostic study, we analyzed a 20% random sample of Medicare fee-for-service beneficiaries and included claims occurring between January 1, 2017, and December 31, 2020.^31,32,33,34,35,36,37,38^ The Stanford University Institutional Review Board approved this study with a waiver of informed consent due to use of administrative claims data. We followed the Standards for Reporting of Diagnostic Accuracy (STARD) reporting guideline. Data analysis was conducted between August 16, 2023, and January 27, 2025. Medicare risk adjustment is prospective, where observed beneficiary demographic characteristics and health conditions from a given year are used to predict spending in the following year. As a result, our analytic cohorts each span 2 calendar years. These cohorts included beneficiaries who were aged 65 years or older, continuously enrolled in Medicare Parts A and B, not enrolled in Medicare Part C, and not dual eligible for Medicaid. Eligibility criteria were based on the largest of the 6 community segments that CMS uses to stratify beneficiaries for risk adjustment. In our study, we included beneficiaries who died in the payment year but excluded beneficiaries who became ineligible for other reasons, such as transitioning to a Part C plan. Additional details are included in the eMethods in Supplement 1.

For each eligible beneficiary, we extracted demographic characteristics from the Master Beneficiary Summary File and diagnoses from inpatient, outpatient, and carrier claims. Demographic characteristics included age, documented sex, race and ethnicity, county, and original reason for Medicare eligibility. Race and ethnicity were based on the enhanced race and ethnicity code that applies the Research Triangle Institute race imputation algorithm (eFigure 1 in Supplement 1).^39^ We used CMS terms for racial and ethnic groups (American Indian or Alaska Native, Asian or Other Pacific Islander, Black, Hispanic, non-Hispanic White), although we used additional group to refer to the group termed *other* by CMS. Beneficiaries in the American Indian or Alaska Native, Asian or Other Pacific Islander, or Black group or an additional group were considered non-Hispanic.

We constructed hierarchical condition categories (HCCs) from *International Statistical Classification of Diseases, Tenth Revision* diagnoses coded in inpatient, outpatient, and carrier claims files. Diagnoses from outpatient and carrier claims files were included after filtering visits by qualifying Healthcare Common Procedure Coding System codes.^40^ We mapped 9474 unique *International Statistical Classification of Diseases, Tenth Revision* diagnosis codes to the 86 HCCs included in CMS’s version 24 risk adjustment software.^41^ Finally, we calculated total annual payments by Medicare from inpatient, outpatient, and carrier claims.

### Fair Spending Targets

We estimated 2 possible sets of fair spending targets, which are prespecified levels of net compensation for minoritized racial and ethnic groups that incorporate fairness goals. Net compensation is the difference between mean predicted spending (from the payment algorithm) and mean observed spending. The first set of spending targets, referred to as disparities-based targets, aimed to capture the additional spending by beneficiaries in minoritized racial and ethnic groups if they had the same health care access and use as non-Hispanic White beneficiaries. Specifically, we fit an ordinary least squares regression to predict spending using data from non-Hispanic White beneficiaries only. The regression included fixed effects for age group, documented sex, number of diagnosed payment-eligible HCCs, whether the beneficiary was first eligible for Medicare based on a disabling condition, and county. Using this regression, we predicted spending for all other racial and ethnic groups. Our disparities-based fair spending targets were set as the difference between mean predicted spending, based on the regression using data from non-Hispanic White beneficiaries only, and mean observed spending for American Indian or Alaska Native, Asian or Other Pacific Islander, Black, and Hispanic racial and ethnic groups.

Our second set of fair spending targets, referred to as 5% targets, added 5% of mean spending across all eligible beneficiaries to net compensation from the baseline regression for minoritized racial and ethnic groups. The 5% targets were based on the current bonus paid to plans with high quality ratings.^8^

In this study, the purpose of our 2 sets of fair spending targets was to examine the potential ability of algorithmic tools to achieve policy-determined fair spending targets. In practice, implemented spending targets should be determined through broad consultation and further research.

### Risk Adjustment Algorithms

First, we established a baseline regression that approximated the least squares regression used by CMS to estimate risk scores for Medicare plan payment risk adjustment. The regression included indicators for 11 combinations of age group and documented sex (age categories: 65-69, 70-74, 75-79, 80-84, 85-89, ≥90 years; documented sex categories: female, male; reference category was female aged 65-69 years), indicators for 86 HCCs, indicators for 6 interactions between HCCs, an indicator for documented female beneficiaries originally eligible for Medicare based on a qualifying disabling condition, and an indicator for documented male beneficiaries originally eligible for Medicare based on a qualifying disabling condition. We also included indicators for county of residence because Medicare payment benchmarks are set by county. In the baseline regression, all coefficients for HCCs, including interactions, were constrained to be nonnegative.

We evaluated 2 algorithms to achieve fair spending targets: constrained regression and a postprocessing algorithm. Both algorithms built on the baseline regression. Constrained regression added constraints that ensured net compensation by minoritized racial and ethnic group matched fair spending targets. Net compensation was not constrained for non-Hispanic White, additional, or unknown racial and ethnic groups. To maintain baseline levels of overall spending, we included a constraint specifying that total predicted spending equaled total observed spending.

As an alternative to constrained regression, we assessed the utility of postprocessing in achieving fair spending targets. After predicting spending using the baseline regression, we added a constant factor equal to the estimated additional payment required to reach fair spending targets for every beneficiary in minoritized racial and ethnic groups. This algorithm would achieve the same levels of in-sample net compensation as constrained regression for groups receiving additional payments. In this case, we ensured that overall spending remained unchanged by subtracting a constant amount from baseline predicted spending for all non-Hispanic White beneficiaries. Details on these risk adjustment algorithms are included in the eMethods in Supplement 1.

### Statistical Analysis

We used 10-fold cross-validation to generate out-of-sample estimates of overall and group-level algorithm performance. Overall performance was measured by mean absolute error and payment system fit. Payment system fit is equivalent to *R*^2^ for the baseline and constrained regressions. Payment system fit for the postprocessing algorithm was equal to 1 minus the residual sum of squares using the postprocessed predicted spending, divided by the total sum of squares. We computed net compensation to evaluate group-level algorithm performance. Finally, since the Medicare data do not include individual-level indicators of socioeconomic status, we evaluated the difference in predicted spending between algorithms by the socioeconomic status domain of the county-level Centers for Disease Control and Prevention and Agency for Toxic Substance and Disease Registry Social Vulnerability Index (SVI).^42^ Higher SVI values reflect greater exposure to socioeconomic factors that can adversely affect health outcomes.

Our main analysis focused on the 2018-2019 cohort because it included the most recent payment year prior to the COVID-19 pandemic for which we had access to data. Sensitivity analyses focused on the 2017-2018 and 2019-2020 cohorts. Additionally, we examined the sensitivity of out-of-sample algorithm performance excluding beneficiaries who died in the payment year.

Analyses were conducted using R version 4.0.3 (R Project for Statistical Computing) and Python version 3.7.17 (Python Software Foundation). We fit all regressions using CVXPY.^43,44^

## Results

Our main analysis included 4 398 035 eligible beneficiaries, of whom fewer than 1% were American Indian or Alaska Native, 2% were Asian or Other Pacific Islander, 6% were Black, 3% were Hispanic, 86% were non-Hispanic White, and 1% were part of an additional group (Table 1). The distribution of Medicare spending was right skewed, with mean (SD) spending of $8345 (18 581) and median spending of $2421 (IQR, $809-$7307) (eFigure 2 in Supplement 1). The mean (SD) age of beneficiaries was 75.2 (7.4) years, 44% were documented as male, and 8% originally qualified for Medicare based on a disabling condition. Among beneficiaries, 42% had zero diagnosed payment-eligible HCCs, 26% had 1 HCC, 14% had 2 HCCs, 14% had 3 to 5 HCCs, and 4% had 6 or more HCCs (eFigure 3 in Supplement 1).

**Table 1.** Sample Size, Characteristics, Observed Spending, and Predicted Spending, Overall and by Race and Ethnicity

Race and ethnicity^a^	Sample size, No.	Observed spending, mean (SD), $	Baseline regression net compensation, $	Age, mean (SD), y	Documented female, %	Documented male, %	Diagnosed HCCs, mean (SD)	Dying in payment year, %	Originally qualifying by disabling condition, %
All	4 398 035	8345 (18581)	0	75.2 (7.4)	55.5	44.5	1.3 (1.8)	3.8	7.6
American Indian or Alaska Native	16 332	9043 (19569)	−448	74.3 (6.9)	57.1	42.9	1.5 (1.9)	3.8	15.0
Asian or Other Pacific Islander	82 367	5868 (17372)	1513	74.3 (7.1)	58.5	41.5	0.9 (1.5)	2.3	4.2
Black	244 408	7734 (19723)	691	74.3 (7.2)	57.0	43.0	1.4 (1.8)	3.5	14.8
Hispanic	151 163	6550 (17432)	1088	74.2 (7.0)	52.2	47.8	1.1 (1.6)	2.8	10.8
Non-Hispanic White	3 785 544	8557 (18581)	−129	75.4 (7.5)	56.0	44.0	1.4 (1.8)	4.0	7.2
Additional group^b^	34 572	7183 (18702)	947	74.6 (6.2)	54.1	45.9	1.2 (1.7)	2.6	11.2
Unknown	83 649	6564 (17522)	67	69.3 (3.2)	35.0	65.0	0.9 (1.4)	1.1	3.0

Abbreviation: HCC, hierarchical condition category.

^a^ We used Centers for Medicare & Medicaid terms for racial and ethnic groups (American Indian or Alaska Native, Asian or Other Pacific Islander, Black, Hispanic, non-Hispanic White). Beneficiaries in the American Indian or Alaska Native, Asian or Other Pacific Islander, or Black group or an additional group were considered non-Hispanic.

^b^ We used additional group to refer to the group termed *other* by the Centers for Medicare & Medicaid Services.

Non-Hispanic White beneficiaries tended to be older than beneficiaries of other racial or ethnic groups (for example, mean [SD] age, 75.4 [7.5] years for Non-Hispanic White beneficiaries compared with mean [SD] age, 74.2 [7.0] years for Hispanic beneficiaries) (Table 1). American Indian or Alaska Native beneficiaries had the highest mean observed spending, followed by non-Hispanic White beneficiaries. Asian or Other Pacific Islander beneficiaries had the lowest mean observed spending, followed by Hispanic beneficiaries. The percentage of American Indian or Alaska Native and Black beneficiaries originally qualifying for Medicare based on a disabling condition was more than twice that of non-Hispanic White beneficiaries.

### Net Compensation and Fair Spending Targets

The baseline regression undercompensated American Indian or Alaska Native (−$448) and non-Hispanic White (−$129) beneficiaries and overcompensated Asian or Other Pacific Islander ($1513), Black ($691), Hispanic ($1088), additional group ($947), and unknown race or ethnicity ($67) beneficiaries (Table 1).

Disparities-based fair spending targets added varying amounts to net compensation from the baseline regression: $300 for American Indian or Alaska Native beneficiaries, $604 for Asian or Other Pacific Islander beneficiaries, $518 for Black beneficiaries, and $513 for Hispanic beneficiaries (Table 2). These targets reflect additional predicted spending if beneficiaries in minoritized racial and ethnic groups had sufficient health care access, such that their spending matched that of non-Hispanic White beneficiaries, conditional on age group, documented sex, number of diagnosed HCCs, originally disabled status, and county of residence (eTable 1 in Supplement 1). Fair spending targets based on 5% of overall mean spending increased net compensation by $417 for minoritized racial and ethnic groups.

**Table 2.** Additional Payments to Reach Fair Spending Targets for Minoritized Racial and Ethnic Groups

Race and ethnicity^a^	Target, $	
	Disparities based^b^	5% of Mean spending^c^
American Indian or Alaska Native	300	417
Asian or Other Pacific Islander	604	417
Black	518	417
Hispanic	513	417

^a^ We used Centers for Medicare & Medicaid terms for racial and ethnic groups (American Indian or Alaska Native, Asian or Other Pacific Islander, Black, Hispanic).

^b^ Disparities-based targets aimed to capture the additional spending by beneficiaries in minoritized racial and ethnic groups if they had the same health care access and use as non-Hispanic White beneficiaries.

^c^ The 5% targets added 5% of mean spending across all eligible beneficiaries to net compensation from the baseline regression for minoritized racial and ethnic groups.

### Algorithm Performance

The baseline regression achieved an out-of-sample mean absolute error of $8359 and payment system fit of 12.7% (Table 3). Achieving fair spending targets using constrained regression or postprocessing resulted in a small reduction in out-of-sample predictive performance. Compared with the baseline regression, payment system fit decreased by less than 0.1 percentage points and mean absolute error increased by at most $21 across both sets of fair spending targets and both algorithms. Out-of-sample net compensation was similar to in-sample baseline net compensation and net compensation for fair spending targets (eTable 2 in Supplement 1).

**Table 3.** Out-of-Sample Algorithm Performance Measures for Baseline Regression, Constrained Regression, and Postprocessing

Algorithm and fair spending target	Payment system fit, %	Mean absolute error, $
Baseline regression	12.7	8359
Disparities based^a^		
Constrained regression	12.6	8380
Postprocessing	12.7	8367
5% of Mean spending^b^		
Constrained regression	12.7	8374
Postprocessing	12.7	8366

^a^ Disparities-based targets aimed to capture the additional spending by beneficiaries in minoritized racial and ethnic groups if they had the same health care access and use as non-Hispanic White beneficiaries.

^b^ The 5% targets added 5% of mean spending across all eligible beneficiaries to net compensation from the baseline regression for minoritized racial and ethnic groups.

**Figure 1.**
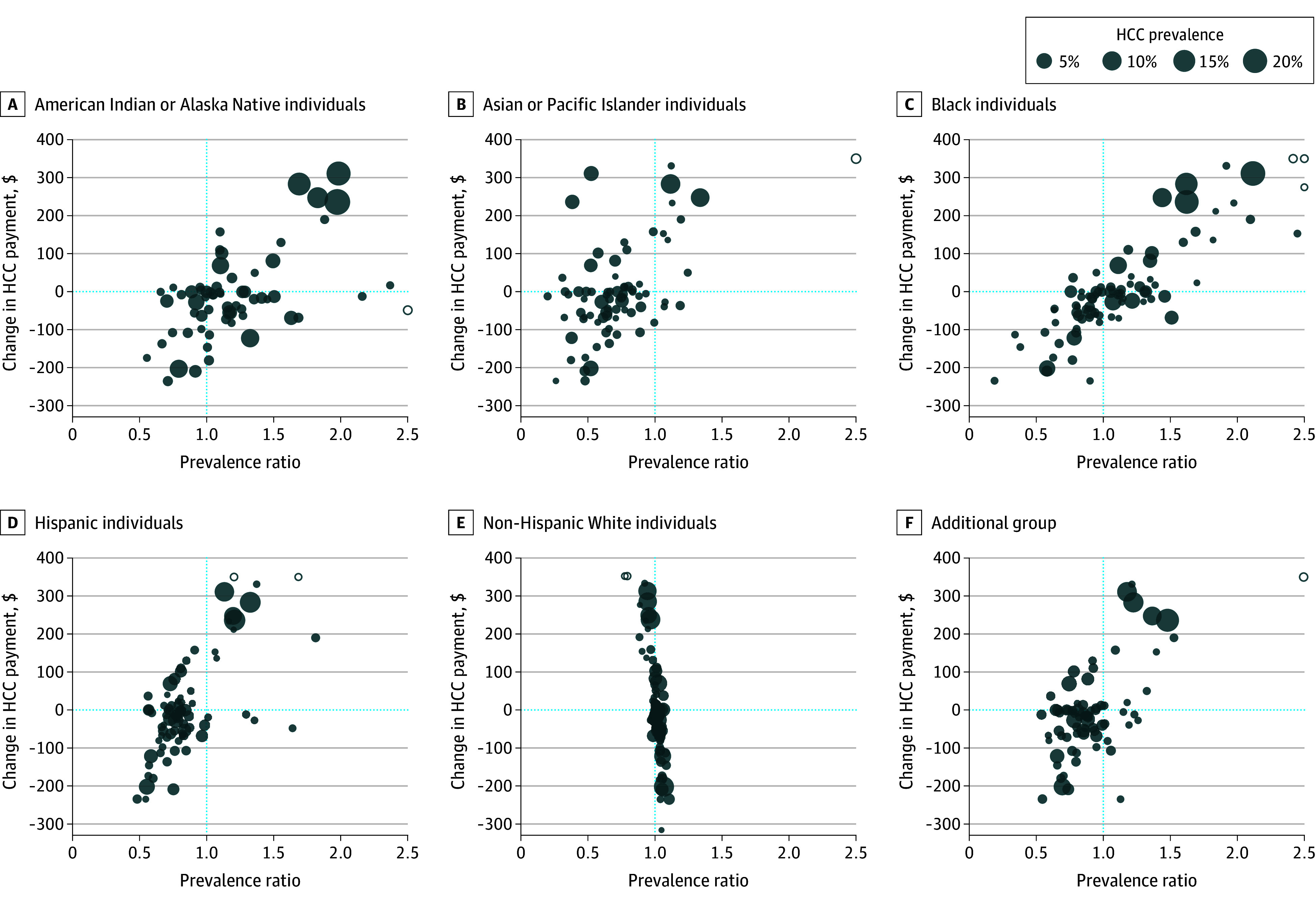
Hierarchical Condition Category (HCC) Prevalence Ratio and Change in HCC Payment Between Baseline and Constrained Regression for Disparities-Based Fair Spending Targets Size of the circles reflects group prevalence of the HCC. Open circles denote top-coded values. Prevalence ratios compare group prevalence with overall prevalence and were top coded at 2.5. Changes in HCC payments were top coded at $350.

The median change in payment for HCCs, comparing the baseline regression with the constrained regression for disparities-based fair spending targets, was −$14 (IQR, −$68 to $30). Constrained regression increased payments for HCCs that were overrepresented among American Indian or Alaska Native, Asian or Other Pacific Islander, Black, and Hispanic beneficiaries, compared with the prevalence of the conditions in the overall sample (Figure 1). Although we did not include constraints on spending for beneficiaries in the additional group, changes in HCC payments with respect to relative prevalence followed a similar pattern to that observed for minoritized racial and ethnic groups.

**Figure 2.**
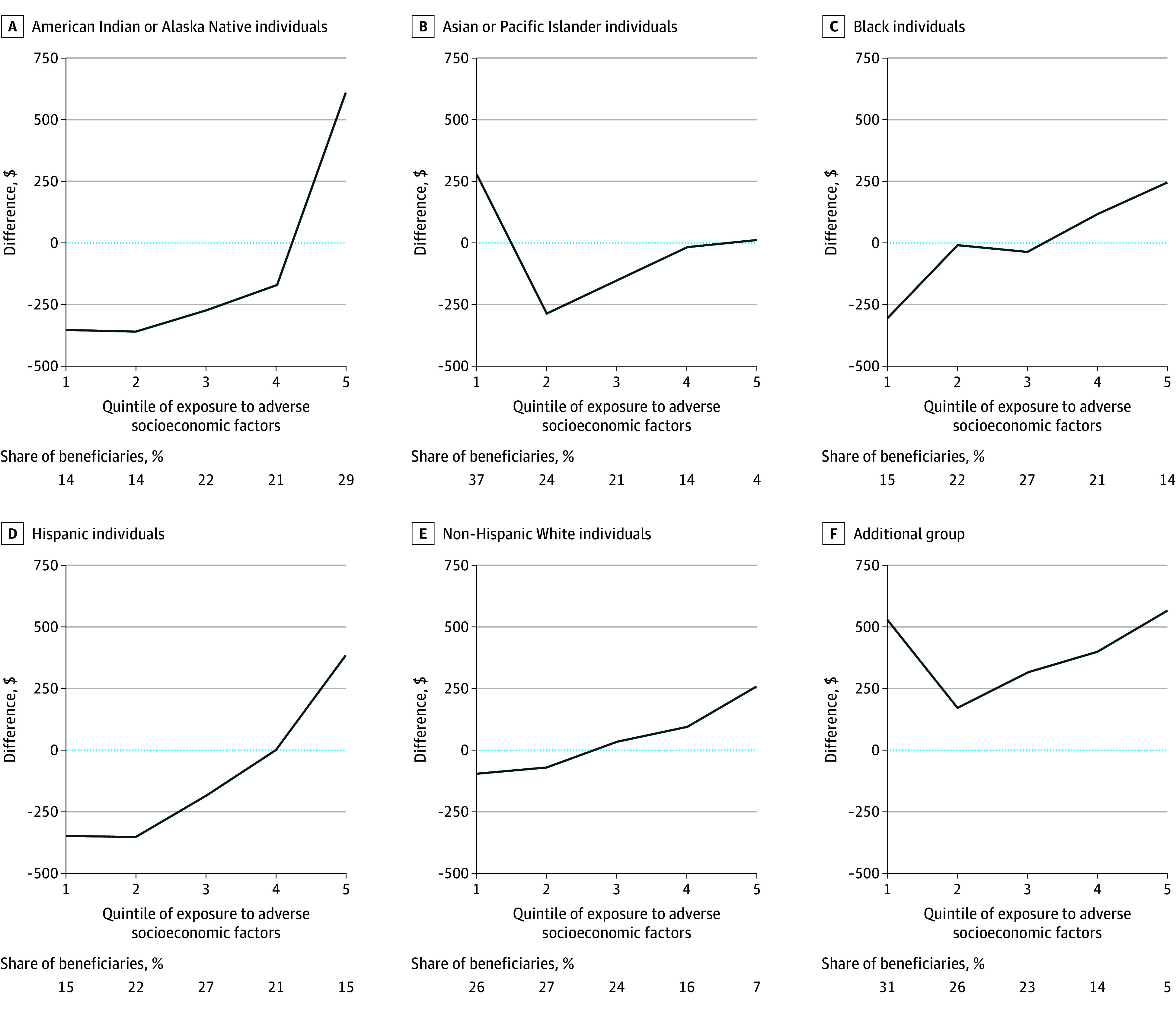
Difference in Predicted Spending Between Constrained Regression and Postprocessing by Racial and Ethnic Group and County-Level Social Vulnerability Index Quintile Percentages denote the proportion of beneficiaries in each racial and ethnic group living in each Social Vulnerability Index quintile, where quintile 1 has the least exposure to adverse socioeconomic factors. We used additional group to refer to the group termed *other* by the Centers for Medicare & Medicaid Services. Beneficiaries in the American Indian or Alaska Native, Asian or Other Pacific Islander, or Black group or an additional group were considered non-Hispanic.

Across SVI quintiles, the postprocessing algorithm increased spending by an equal amount for beneficiaries of the same minoritized racial and ethnic group and decreased spending by an equal amount for non-Hispanic White beneficiaries. In contrast, differences between mean spending from the baseline regression and mean spending from the constrained regression varied by racial and ethnic group and county SVI quintile. Predicted spending from the constrained regression, compared with the postprocessing algorithm, was generally greater for beneficiaries living in counties with more exposure to adverse socioeconomic factors across racial and ethnic groups (Figure 2).

### Sensitivity Analyses

In sensitivity analyses focusing on the 2017-2018 and 2019-2020 cohorts, our algorithmic framework yields largely stable results over time (eTables 3-6 in Supplement 1). Payment system fit differences between the baseline regression, constrained regression, and postprocessing algorithm were negligible in alternative cohorts, similar to those observed in the main analysis (eTable 7 in Supplement 1). Excluding beneficiaries who died in the payment year also resulted in negligible differences between algorithms. Trends in predicted spending by racial and ethnic group and county SVI quintile for alternative cohorts were similar to those documented in the main analysis (eFigures 4 and 5 in Supplement 1).

## Discussion

This study developed 2 algorithms, constrained regression and postprocessing, to achieve fairness goals across multiple minoritized racial and ethnic groups in Medicare risk adjustment. Both algorithms reached fair spending targets without compromising overall fit compared with the baseline regression. Notably, both algorithms represent feasible extensions of the regression currently used to estimate risk scores, which supports algorithm flexibility, transparency, interpretability, and potential for implementation.

Whereas postprocessing directly changes payments for specified groups, constrained regression changes payments for health conditions, which can affect all groups. As a result, for our fair spending targets, we found that constrained regression can achieve more widespread equity-enhancing changes in payments compared with postprocessing. This is a desirable feature when leveraging payment reform to broadly mitigate health disparities, to the extent that social and structural forces result in the clustering of certain health conditions among marginalized groups.

Consistent with prior literature, we found that the current risk adjustment algorithm undercompensates American Indian or Alaska Native and non-Hispanic White Medicare beneficiaries and overcompensates Asian or Other Pacific Islander, Black, and Hispanic beneficiaries.^28^ Observed spending is lowest among overcompensated racial and ethnic groups, likely as a result of health care access barriers leading to lower use.^24^ Although most minoritized racial and ethnic groups are already overcompensated, our disparities-based fair spending targets suggest that resource reallocation through the current risk adjustment algorithm may be insufficient to fully mitigate disparities. From a health equity perspective, increased payments for marginalized groups may be needed to reduce inequalities in access and outcomes. If budget neutrality were not required to implement fair spending targets, estimated total Medicare Advantage spending would increase by less than 2%.

Both algorithms evaluated in our study achieve fair spending targets for multiple minoritized racial and ethnic groups with negligible reduction in overall payment system fit. This aligns with prior work on fair regression, contributing to a mounting case for payment system reform that aims to achieve the dual goals of equity and efficiency.^11,14,15,16,18,19,28^ The Netherlands has applied constrained regression to ensure fair payment for individuals with multiple chronic illnesses.^45^ Two crucial policy features must be determined before such reform is implemented: the fair spending targets and the system for ensuring that additional payments are effectively used to address drivers of disparities.

### Limitations

Our fair spending targets allow us to assess the utility of algorithmic tools. Fair spending targets included in any payment reform policy should be determined through broad consultation and additional research. Further examination of potential consequences for groups included and not included in fairness objectives is necessary. Additionally, existing payment systems and our analysis both rely heavily on diagnosed health conditions. Underdiagnosis of health conditions among minoritized racial and ethnic groups would result in lower predicted spending compared with that expected if all racial and ethnic groups experienced equal rates of diagnosis. As a result, our estimates of disparities are likely smaller than those estimated if we were to adjust for beneficiaries’ true health status. Future research should focus on evaluating and mitigating the effects of differential rates of diagnosis in payment policy.

Strategic responses by insurers, the extent to which additional payments would affect beneficiaries’ experiences, and the potential for such payments to mitigate disparities in health care access and outcomes were not included in our study. Other policy levers would need to be paired with algorithmic changes to the payment system to increase the likelihood that reform yields desired outcomes. Notably, health care disparities are the result of many long-standing and interconnected social and structural forces.^29^ Algorithmic changes and market forces alone cannot eliminate disparities, but algorithms can be designed to support and contribute to a more equitable system.^46^

We did not have access to self-reported race and ethnicity. Despite the Research Triangle Institute enhanced race and ethnicity variable having better validity than the variable based directly on Social Security Administration documentation, misclassification of race and ethnicity remains a persistent problem.^47^ Furthermore, health spending and outcome disparities exist across multiple social and structural dimensions. Although our analysis focuses on increasing spending for minoritized racial and ethnic groups, additional spending may be warranted to mitigate disparities for other groups. Finally, our data include only fee-for-service claims, so we could not evaluate differences in health status, spending, use, and outcomes between beneficiaries enrolled in traditional Medicare vs Medicare Advantage.

## Conclusions

The results of this study support the idea that feasible changes to the Medicare risk adjustment algorithm can achieve fairness goals for multiple minoritized racial and ethnic groups, with a negligible effect on overall payment system fit. Payment system reform that incorporates fair spending targets should be considered by policymakers aiming to address health care disparities.

## Notes

Supplement 1. **eMethods.** Eligibility and Algorithm Details **eFigure 1.** Difference Between the Social Security Administration Race/Ethnicity Label and the Research Triangle Institute’s Enhanced Race/Ethnicity Label **eFigure 2.** Distribution of Spending Among the 2018-2019 Cohort **eFigure 3.** Distribution of the Number of Payment-Eligible Hierarchical Condition Categories Among the 2018-2019 Cohort **eTable 1.** Regression Coefficients for the Disparities-Based Targets **eTable 2.** Out-of-Sample Net Compensation by Algorithm, Spending Target, and Racial/Ethnic Group for the 2018-2019 Cohort **eTable 3**. Sample Size, Characteristics, Observed Spending, and Predicted Spending, Overall and by Race/Ethnicity for the 2017-2018 Cohort **eTable 4**. Sample Size, Characteristics, Observed Spending, and Predicted Spending, Overall and by Race/Ethnicity for the 2019-2020 Cohort **eTable 5**. Observed Spending and Net Compensation by Algorithm, Spending Target, and Racial/Ethnic Group for the 2017-2018 Cohort **eTable 6**. Observed Spending and Net Compensation by Algorithm, Spending Target, and Racial/Ethnic Group for the 2019-2020 Cohort **eTable 7.** Payment System Fit Across Algorithms, Spending Targets, and Cohorts **eFigure 4**. Difference in Predicted Spending Between Constrained Regression and Post-Processing by Racial/Ethnic Group and County-Level Social Vulnerability Index Quintile for the 2017-2018 Cohort **eFigure 5**. Difference in Predicted Spending Between Constrained Regression and Post-Processing by Racial/Ethnic Group and County-Level Social Vulnerability Index Quintile for the 2019-2020 Cohort
eReferences

Supplement 2.
Data Sharing Statement
