# Supplementary material for: Algorithms to Improve Fairness in Medicare Risk Adjustment
Source: JAMA Health Forum. 2025 Aug 29;6(8):e252640. doi: 10.1001/jamahealthforum.2025.2640 (PMC12397885; doi:10.1001/jamahealthforum.2025.2640)
Supplement: Supplement 1. — eMethods. Eligibility and Algorithm Details eFigure 1. Difference Between the Social Security Administration Race/Ethnicity Label and the Research Triangle Institute’s Enhanced Race/Ethnicity Label eFigure 2. Distribution of Spending Among the 2018-2019 Cohort eFigure 3. Distribution of the Number of Payment-Eligible Hierarchical Condition Categories Among the 2018-2019 Cohort eTable 1. Regression Coefficients for the Disparities-Based Targets eTable 2. Out-of-Sample Net Compensation by Algorithm, Spending Target, and Racial/Ethnic Group for the 2018-2019 Cohort eTable 3. Sample Size, Characteristics, Observed Spending, and Predicted Spending, Overall and by Race/Ethnicity for the 2017-2018 Cohort eTable 4. Sample Size, Characteristics, Observed Spending, and Predicted Spending, Overall and by Race/Ethnicity for the 2019-2020 Cohort eTable 5. Observed Spending and Net Compensation by Algorithm, Spending Target, and Racial/Ethnic Group for the 2017-2018 Cohort eTable 6. Observed Spending and Net Compensation by Algorithm, Spending Target, and Racial/Ethnic Group for the 2019-2020 Cohort eTable 7. Payment System Fit Across Algorithms, Spending Targets, and Cohorts eFigure 4. Difference in Predicted Spending Between Constrained Regression and Post-Processing by Racial/Ethnic Group and County-Level Social Vulnerability Index Quintile for the 2017-2018 Cohort eFigure 5. Difference in Predicted Spending Between Constrained Regression and Post-Processing by Racial/Ethnic Group and County-Level Social Vulnerability Index Quintile for the 2019-2020 Cohort eReferences [file jamahealthforum-e252640-s001.pdf]

Supplemental Online Content

Reitsma MB, McGuire TG, Rose S. Algorithms to improve fairness in Medicare risk adjustment. *JAMA Health Forum*. Published online August 29, 2025. doi: 10.1001/jamahealthforum.2025.2640

eMethods. Eligibility and Algorithm Details

eFigure 1. Difference Between the Social Security Administration Race/Ethnicity Label and the Research Triangle Institute's Enhanced Race/Ethnicity Label

eFigure 2. Distribution of Spending Among the 2018-2019 Cohort

eFigure 3. Distribution of the Number of Payment-Eligible Hierarchical Condition Categories Among the 2018-2019 Cohort

eTable 1. Regression Coefficients for the Disparities-Based Targets

eTable 2. Out-of-Sample Net Compensation by Algorithm, Spending Target, and Racial/Ethnic Group for the 2018-2019 Cohort

eTable 3. Sample Size, Characteristics, Observed Spending, and Predicted Spending, Overall and by Race/Ethnicity for the 2017-2018 Cohort

eTable 4. Sample Size, Characteristics, Observed Spending, and Predicted Spending, Overall and by Race/Ethnicity for the 2019-2020 Cohort

eTable 5. Observed Spending and Net Compensation by Algorithm, Spending Target, and Racial/Ethnic Group for the 2017-2018 Cohort

eTable 6. Observed Spending and Net Compensation by Algorithm, Spending Target, and Racial/Ethnic Group for the 2019-2020 Cohort

eTable 7. Payment System Fit Across Algorithms, Spending Targets, and Cohorts

eFigure 4. Difference in Predicted Spending Between Constrained Regression and Post-Processing by Racial/Ethnic Group and County-Level Social Vulnerability Index Quintile for the 2017-2018 Cohort

eFigure 5. Difference in Predicted Spending Between Constrained Regression and Post-Processing by Racial/Ethnic Group and County-Level Social Vulnerability Index Quintile for the 2019-2020 Cohort

eReferences

This supplemental material has been provided by the authors to give readers additional information about their work.

© 2025 Reitsma MB et al. *JAMA Health Forum*.

eMethods. Eligibility and algorithm details.

Eligibility

We created three analytic cohorts that span two calendar years: 2017-2018, 2018-2019, and 2019-2020. For each two-year window, we identified eligible beneficiaries using the Master Beneficiary Summary File (MBSF) Base records. Beneficiaries were eligible to be included in our primary analyses if they met the following criteria:

- Aged 65 years or older by the end of the first calendar year.
- Enrolled in Medicare Parts A and B for all 12 months in the first year and did not die in the first year.
- Enrolled in Medicare Parts A and B for all 12 months in the second year, or were enrolled in Medicare Parts A and B until their month of death in the second year.
- Not enrolled in Medicare Part C at any point in the first or second calendar year.
- Not dual eligible for Medicaid at any point in the first or second calendar year.

We were not able to separate community from institutional populations. Based on 2014-2015 sample counts reported in the 2021 CMS Report to Congress, the institutional population accounts for only three percent of the total population of fee-for-service beneficiaries.[1]

Algorithm Details

Notation

Our risk adjustment algorithms aimed to predict spending Y in year $t+1$ for beneficiary i as a function of demographic characteristics D, diagnosed health conditions H, and county of residence C in year t. The vector C included 2,943 county indicators, as a result of grouping counties within states that had fewer than the tenth percentile of eligible beneficiaries.

Baseline Regression

First, we approximated the least squares regression approach used by CMS to estimate the risk scores for Medicare Advantage plan payment risk adjustment:

$$Y_i = \alpha + \beta D_i + \gamma H_i + \delta C_i,$$

where γ is constrained to be non-negative. We fit this regression by minimizing the square root of the sum of squared residuals, which yields an equivalent result to minimizing the sum of squared residuals.[2]

We deviated from the CMS Version 24 formula by grouping beneficiaries aged 95+ with beneficiaries aged 90-94 due to the small sample sizes for the 95+ group. We also excluded the indicators capturing the number of HCCs.

Constrained Regression and Post-Processing for Fair Spending

We evaluated two approaches to achieve fair spending targets: constrained regression and post-processing. Fairness constrained regression included additional constraints on net compensation by racial/ethnic group. We defined group-level net compensation as the mean difference between predicted spending, $\hat{Y}$, and observed spending for beneficiaries p in racial/ethnic group $j \in$ {American Indian/Alaska Native, Asian/Pacific Islander, Black, Hispanic}:

$$G_j = \frac{1}{n_j}\sum_{p \in j}\left(\hat{Y}_{p,j} - Y_{p,j}\right),$$

where n_j was the number of beneficiaries in group j and the set of sets j is J.

Similar to the baseline regression, we fit the constrained regression by minimizing the square root of the sum of squared residuals:

$$\underset{\alpha,\beta,\gamma,\delta}{\text{minimize}}\{||Y_i - (\alpha + \beta D_i + \gamma H_i + \delta C_i)||_2\},$$

subject to: $\gamma \geq 0$, $G_j = F_j$, and $\sum_i \hat{Y}_i = \sum_i Y_i$, where F_j is the group-specific fair spending target.

For our post-processing algorithm, after predicting spending in the baseline regression, we added a constant group-specific payment to beneficiaries in minoritized racial/ethnic groups such that net compensation after post-processing equaled fair spending targets: $\hat{Y}^*_{p,j} = \hat{Y}_{p,j} + (F_j - G_j)$. To ensure that total spending remained unchanged, we subtracted a constant payment from all n_w beneficiaries in the non-Hispanic white group w, indexed by k: $\hat{Y}^*_k = \hat{Y}_k - \frac{\sum_{j \in J} \sum_{p \in j} \left(\hat{Y}^*_{p,j} - \hat{Y}_{p,j}\right)}{n_w}$.

© 2025 Reitsma MB et al. *JAMA Health Forum*.

eFigure 1. Difference between the Social Security Administration race/ethnicity label and the Research Triangle Institute's enhanced race/ethnicity label.

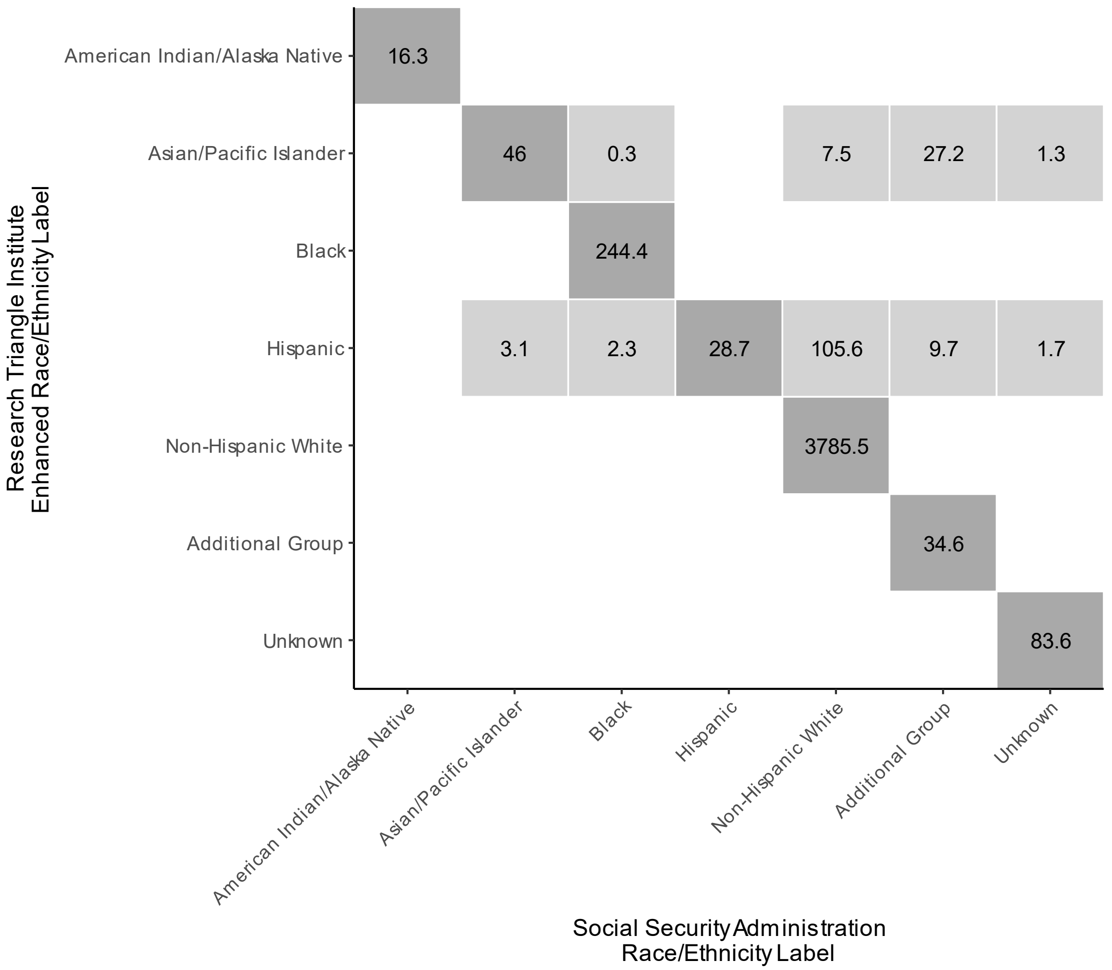

Note: Numbers are thousands of beneficiaries.

© 2025 Reitsma MB et al. *JAMA Health Forum*.

eFigure 2. Distribution of spending among the 2018-2019 cohort.

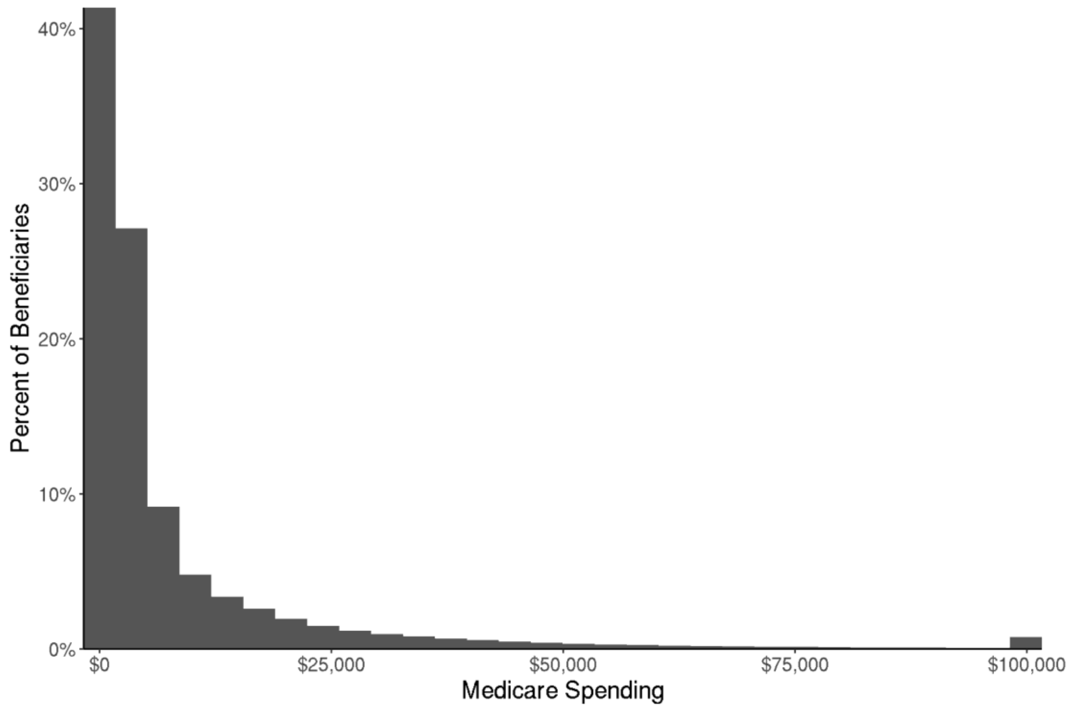

Note: Spending is top-coded at $100,000 in the figure due to long right tail.

© 2025 Reitsma MB et al. *JAMA Health Forum*.

eFigure 3. Distribution of the number of payment-eligible hierarchical condition categories among the 2018-2019 cohort.

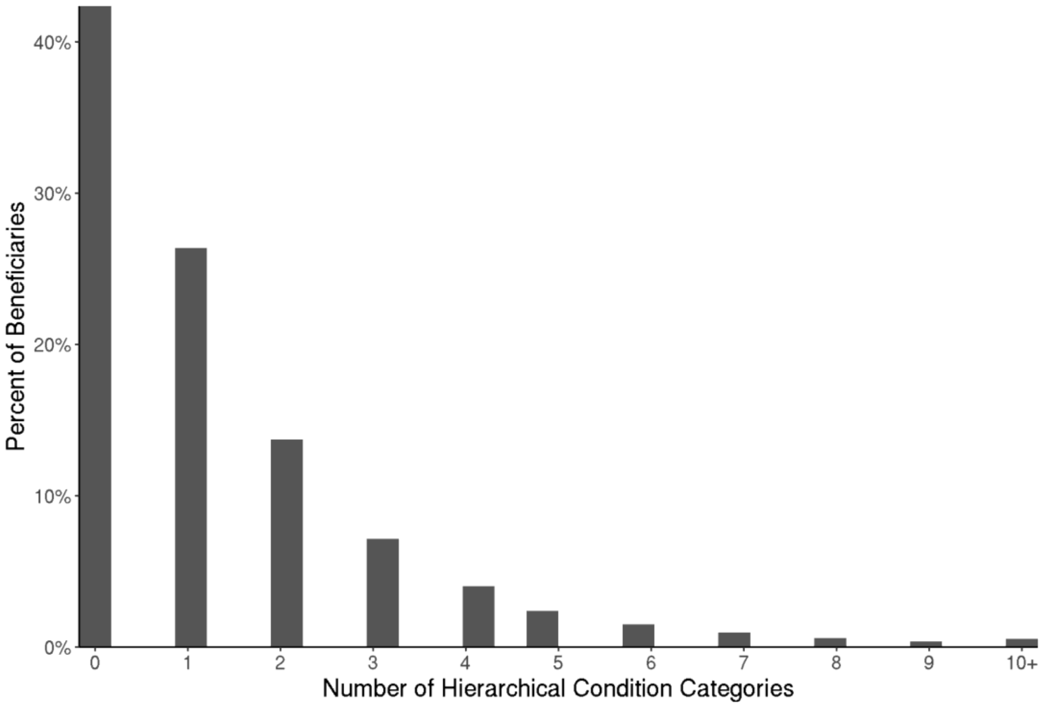

Note: Number of Hierarchical Condition Categories is top-coded at 10 in figure due to long right tail.

© 2025 Reitsma MB et al. *JAMA Health Forum*.

eTable 1. Regression coefficients for the disparities-based targets.

Variable	Coefficient
First Eligible for Medicare Based on a Disabling Condition	$1,519
Number of Diagnosed Payment-Eligible HCCs	$2,966
Female 65-69	$0
Female 70-74	$509
Female 75-79	$974
Female 80-84	$875
Female 85-89	$501
Female 90+	-$566
Male 65-69	-$123
Male 70-74	$616
Male 75-79	$1,602
Male 80-84	$1,700
Male 85-89	$1,381
Male 90+	$379

Note: Coefficients for counties are not reported.

© 2025 Reitsma MB et al. *JAMA Health Forum*.

eTable 2. Out-of-sample net compensation by algorithm, spending target, and racial/ethnic group for the 2018-2019 cohort.

Race/Ethnicity	Baseline Regression	Disparities: Constrained Regression	Disparities: Post-Processing	Five Percent: Constrained Regression	Five Percent: Post-Processing
American Indian/Alaska Native	-$442	-$148	-$142	-$31	-$25
Asian/Pacific Islander	$1,513	$2,116	$2,117	$1,929	$1,930
Black	$692	$1,207	$1,209	$1,107	$1,109
Hispanic	$1,088	$1,600	$1,602	$1,504	$1,506
Non-Hispanic White	-$129	-$201	-$198	-$186	-$184
Additional Group	$947	$1,318	$947	$1,211	$947
Unknown	$67	$67	$67	$62	$67

eTable 3. Sample size, characteristics, observed spending, and predicted spending, overall and by race/ethnicity for the 2017-2018 cohort.

Race/Ethnicity	Sample Size	Mean Observed Spending	Baseline Regression Net Compensation	Mean Age	Percent Documented Male	Mean Number of Diagnosed HCCs	Percent Dying in the Payment Year	Percent Originally Qualifying by Disabling Condition
All	4,364,834	$7,977	$0	75.2	44.5%	1.3	3.9%	7.7%
American Indian/Alaska Native	15,928	$8,658	-$370	74.3	43.1%	1.5	3.9%	15.0%
Asian/Pacific Islander	79,036	$5,590	$1,433	74.3	42.1%	0.9	2.5%	4.2%
Black	245,457	$7,306	$734	74.3	43.1%	1.4	3.6%	14.9%
Hispanic	150,123	$6,268	$1,040	74.2	48.0%	1.1	3.0%	10.9%
Non-Hispanic White	3,767,527	$8,180	-$125	75.5	44.0%	1.3	4.1%	7.2%
Additional Group	33,327	$6,933	$822	74.4	46.1%	1.1	2.8%	11.5%
Unknown	73,436	$6,182	$16	68.9	65.7%	0.8	1.2%	3.1%

© 2025 Reitsma MB et al. *JAMA Health Forum*.

eTable 4. Sample size, characteristics, observed spending, and predicted spending, overall and by race/ethnicity for the 2019-2020 cohort.

Race/Ethnicity	Sample Size	Mean Observed Spending	Baseline Regression Net Compensation	Mean Age	Percent Documented Male	Mean Number of Diagnosed HCCs	Percent Dying in the Payment Year	Percent Originally Qualifying by Disabling Condition
All	4,443,148	$7,841	$0	75.2	44.3%	1.4	4.2%	7.5%
American Indian/Alaska Native	16,238	$8,907	-$526	74.6	42.3%	1.6	5.1%	15.1%
Asian/Pacific Islander	85,688	$5,296	$1,557	74.3	41.0%	1.0	2.7%	4.2%
Black	240,205	$7,503	$503	74.4	42.7%	1.4	4.3%	14.5%
Hispanic	152,745	$6,178	$967	74.3	47.6%	1.1	3.6%	10.6%
Non-Hispanic White	3,819,616	$8,028	-$114	75.4	43.9%	1.4	4.4%	7.0%
Additional Group	35,453	$6,645	$947	74.9	45.8%	1.2	3.1%	10.8%
Unknown	93,203	$6,354	$91	69.8	64.3%	0.9	1.4%	2.9%

© 2025 Reitsma MB et al. *JAMA Health Forum*.

eTable 5. Observed spending and net compensation by algorithm, spending target, and racial/ethnic group for the 2017-2018 cohort.

Race/Ethnicity	Observed	Baseline Regression	Disparities: Constrained Regression	Disparities: Post-Processing	Five Percent: Constrained Regression	Five Percent: Post-Processing
American Indian/Alaska Native	$8,658	-$370	-$146	-$146	$29	$29
Asian/Pacific Islander	$5,590	$1,433	$2,032	$2,032	$1,832	$1,832
Black	$7,306	$734	$1,235	$1,235	$1,133	$1,133
Hispanic	$6,268	$1,040	$1,667	$1,667	$1,439	$1,439
Non-Hispanic White	$8,180	-$125	-$200	-$196	-$179	-$177
Additional Group	$6,933	$822	$1,174	$822	$1,062	$822
Unknown	$6,182	$16	$26	$16	$18	$16

© 2025 Reitsma MB et al. *JAMA Health Forum*.

eTable 6. Observed spending and net compensation by algorithm, spending target, and racial/ethnic group for the 2019-2020 cohort.

Race/Ethnicity	Observed	Baseline Regression	Disparities: Constrained Regression	Disparities: Post-Processing	Five Percent: Constrained Regression	Five Percent: Post-Processing
American Indian/Alaska Native	$8,907	-$526	-$369	-$369	-$134	-$134
Asian/Pacific Islander	$5,296	$1,557	$2,076	$2,076	$1,949	$1,949
Black	$7,503	$503	$919	$919	$895	$895
Hispanic	$6,178	$967	$1,446	$1,446	$1,359	$1,359
Non-Hispanic White	$8,028	-$114	-$175	-$172	-$167	-$165
Additional Group	$6,645	$947	$1,276	$947	$1,204	$947
Unknown	$6,354	$91	$84	$91	$79	$91

© 2025 Reitsma MB et al. *JAMA Health Forum*.

eTable 7. Payment system fit across algorithms, spending targets, and cohorts.

**Algorithm and Fair Spending Target**	**2017-2018 Cohort**	**2018-2019 Cohort**	**2019-2020 Cohort**
Baseline Regression	12.3%	12.7%	11.5%
Constrained Regression Disparities	12.2%	12.6%	11.4%
Post-Processing Disparities	12.3%	12.7%	11.4%
Constrained Regression Five Percent	12.3%	12.7%	11.4%
Post-Processing Five Percent	12.3%	12.7%	11.4%

© 2025 Reitsma MB et al. *JAMA Health Forum*.

eFigure 4. Difference in predicted spending between constrained regression and post-processing by racial/ethnic group and county-level Social Vulnerability Index quintile for the 2017-2018 cohort.

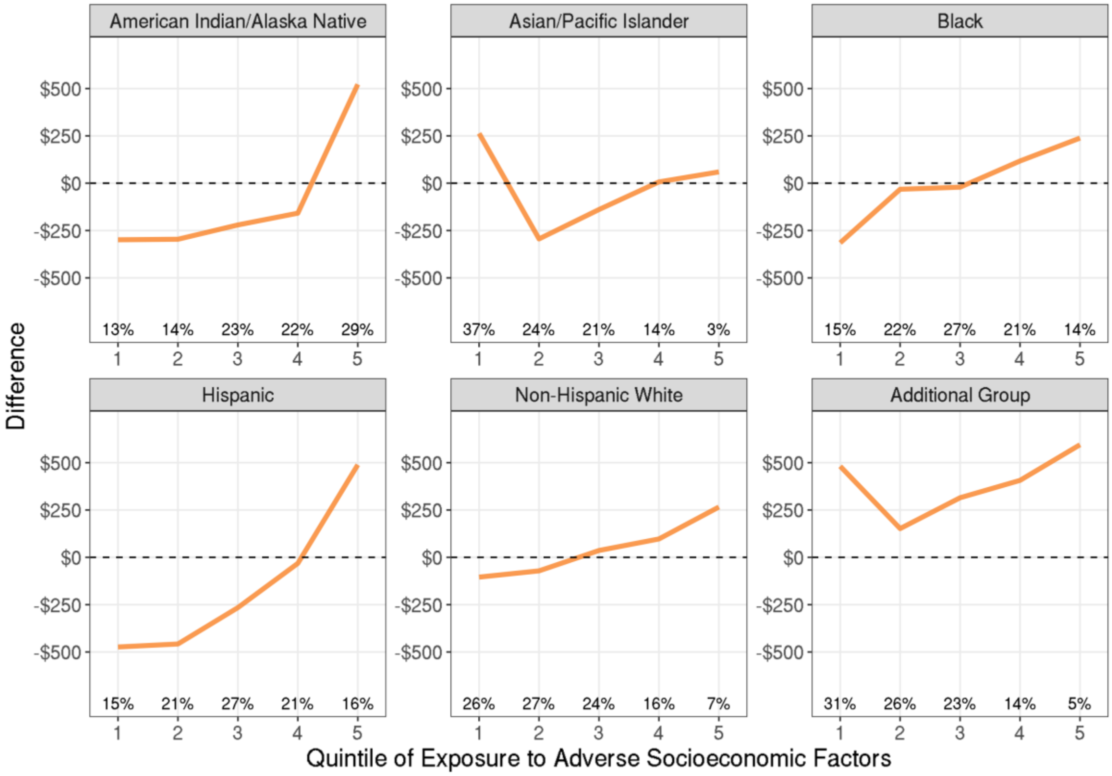

Note: Percentages denote the share of beneficiaries in each racial/ethnic group living in each Social Vulnerability Index quintile, where quintile 1 has the least exposure to adverse socioeconomic factors.

© 2025 Reitsma MB et al. *JAMA Health Forum*.

eFigure 5. Difference in predicted spending between constrained regression and post-processing by racial/ethnic group and county-level Social Vulnerability Index quintile for the 2019-2020 cohort.

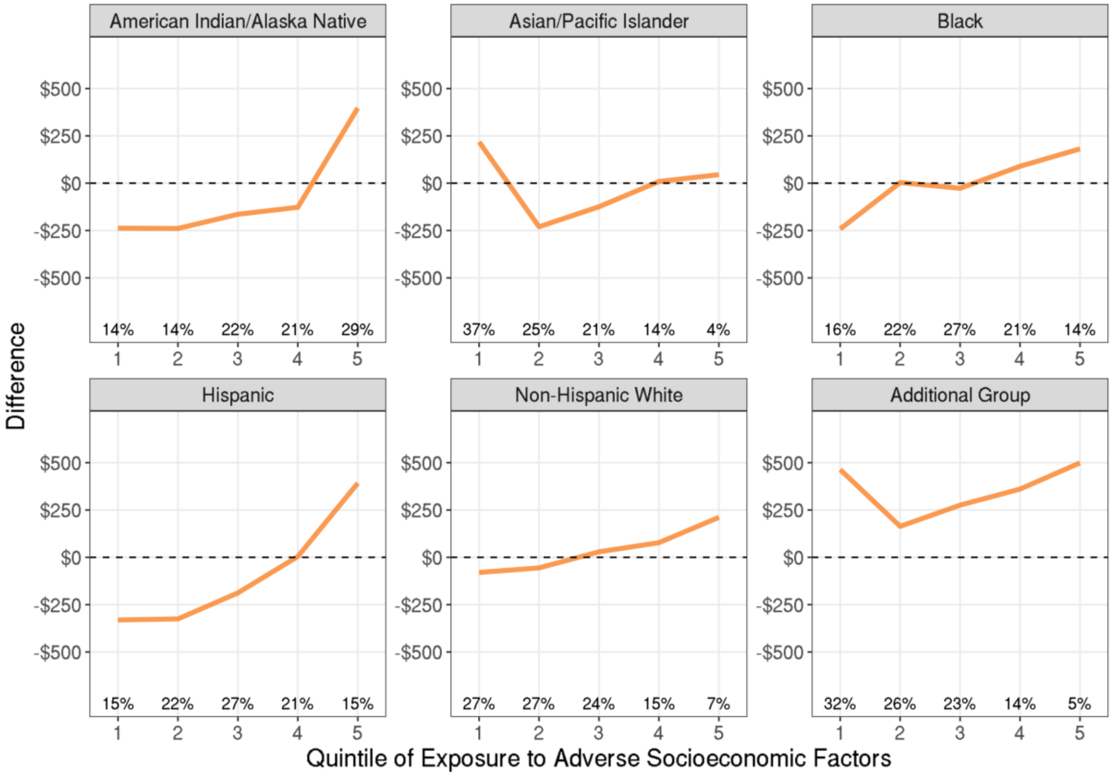

Note: Percentages denote the share of beneficiaries in each racial/ethnic group living in each Social Vulnerability Index quintile, where quintile 1 has the least exposure to adverse socioeconomic factors.

© 2025 Reitsma MB et al. *JAMA Health Forum*.

eReferences.

1. *Report to Congress: Risk Adjustment in Medicare Advantage*. 2021. https://www.cms.gov/files/document/report-congress-risk-adjustment-medicare-advantage-december-2021.pdf
2. Advanced topics CVX users' guide. https://cvxr.com/cvx/doc/advanced.html#eliminating-quadratic-forms

© 2025 Reitsma MB et al. *JAMA Health Forum*.
