# Supplementary material for: Algorithms to Improve Fairness in Medicare Risk Adjustment
Source: JAMA Health Forum. 2025 Aug 29;6(8):e252640. doi: 10.1001/jamahealthforum.2025.2640 (PMC12397885; doi:10.1001/jamahealthforum.2025.2640)
Supplement: Supplement 2. — Data Sharing Statement [file jamahealthforum-e252640-s002.pdf]

Data Sharing Statement

Reitsma. Algorithms to Improve Fairness in Medicare Risk Adjustment. *JAMA Health Forum*. Published August 29, 2025. doi:10.1001/jamahealthforum.2025.2640

Data
Data available: No

Additional Information
Explanation for why data not available: Medicare data contain protected health information and cannot be accessed without IRB approval and a Research Identifiable File (RIF) Data Use Agreement with the Centers for Medicare & Medicaid Services. As a result, no data are made available, but code that allows for replication by researchers with access to the Medicare RIF data is available online at: https://github.com/StanfordHPDS/medicare_fair_risk_adjustment.
